# Adherence to a western dietary pattern and risk of invasive ductal and lobular breast carcinomas: a case–control study

**DOI:** 10.1038/s41598-022-09725-5

**Published:** 2022-04-07

**Authors:** Elahe Foroozani, Ali Akbari, Sasan Amanat, Nastaran Rashidi, Dariush Bastam, Shima Ataee, Golnaz Sharifnia, Mohammad Faraouei, Mostafa Dianatinasab, Hassan Safdari

**Affiliations:** 1 School of Physical and Occupational Therapy, McGill University, Montreal, Canada; 2 The College of Health Sciences, The University of Memphis, Memphis, USA; 3 Department of Nutrition, School of Health, Larestan University of Medical Sciences, Larestan, Iran; 4 Department of Physiology, School of Medicine, Isfahan University of Medical Sciences, Isfahan, Iran; 5 Medical School, Yasuj University of Medical Sciences, Yasuj, Iran; 6 Behbahan Faculty of Medical Sciences, Behbahan, Iran; 7 Department of Epidemiology, School of Public Health, Shahid Sadoughi University of Medical Sciences, Yazd, Iran; 8 Department of Epidemiology, Faculty of Public Health, Shiraz University of Medical Sciences, Shiraz, Iran; 9 Department of Complex Genetics and Epidemiology, School of Nutrition and Translational Research in Metabolism, Maastricht University, Universiteitssingel 40 (Room C5.570), 6229 ER Maastricht, The Netherlands; 10 Department of Anaesthesiology and Perioperative Medicine, Tufts University School of Medicine, Boston, USA

**Keywords:** Cancer, Diseases, Oncology, Risk factors

## Abstract

Little is known about the role of diet in the risk of invasive ductal carcinoma (IDC) and invasive lobular carcinoma (ILC) of the breast, the most common histological subtypes of breast cancer (BC). This is because, the majority of studies on the association of diet and the risk of BC are focused on single food items, and studies considering the overall diet in terms of dietary patterns are limited. Also, the potential heterogeneity in the impact of Western diet (WD) on histological subtypes of BC is not established. This, the age-frequency-matched case–control study included 1009 incident BC cases and 1009 healthy controls. The required data was obtained from the patients’ medical files and interviews using a previously validated researcher-designed questionnaire for collecting data on socio-economic and anthropometric statuses and a valid food frequency questionnaire (FFQ) to measure the participants’ dietary intake. We used multinomial logistic regression, and odds ratios (ORs) with 95% confidence intervals (CIs) were calculated. A positive and significant association was observed between higher adherence to a WD and risk of IDC (OR comparing highest with the lowest tertile: 2.45, 95% CI 1.88, 3.17; p-trend < 0.001), whereas no significant association was observed between adherence to the WD and the risk of ILC (OR comparing highest with the lowest tertile: 1.63, 95% CI 0.63, 3.25) (p for heterogeneity = 0.03). The results of an analysis stratified by menopausal status suggested a similar pattern. We provided evidence that adherence to a WD raises the risk of IDC, but not ILC, suggesting different etiological mechanisms for IDC and ILC.

## Introduction

Accounting for about 25% of all female malignancies and 1 million new cases worldwide annually, breast cancer (BC) is a common neoplasm among women. Concerning developed countries, BC is even more common as it is accounted for 27% of all cancers among women from these countries^1^. However, the rate of age-related mortality from BC is decreasing in high-income countries, whereas the mortality is increasing in lower-income countries^2^. BC is also the most common cancer among Iranian women, and the mean age at diagnosis is significantly lower compared with the western countries^3,4^. As a result, the vast majority of BC cases among Iranian women are premenopausal^3^.

Breast malignancies may occur in the mammary glands with a wide variety of morphological, immunohistochemical and histopathological subtypes and different clinical presentations and outcomes^5^. For example, clinical and epidemiologic studies suggested that the histopathological subtypes of BC differ in terms of behaviour, risk factors and even response to treatment^6,7^. Among different types of BC, invasive ductal (IDC) and lobular carcinomas (ILC) are the first and second most common breast carcinomas (75% and 15% of all breast malignant tumours, respectively). Also, the locations of these tumours are different as the IDC mostly starts in the cells that line a milk duct in the breast and ILC starts in the milk-producing glands (lobules). It is also reported that these two subtypes have distinguishing clinical, molecular and pathologic features^7,8^. For example, it has been suggested that, prognosis of ILC was significantly worse compared to IDC^9^. Also, compared to IDC, ILC patients tend to be at risk for distant recurrence for longer than 5–10 years period^10^ and ILC is considered to be less chemo-sensitive for either adjuvant or neoadjuvant chemotherapy compared to IDC^9^. However, limited evidence is available on the risk factors of ILC and IDC, and very few epidemiological studies have examined the heterogeneity in the factors associated with these subtypes of BC, with no attention to these two BC subtypes^7,11,12^. Also, evidence on this topic from less-developed countries is limited^13^.

With regard to the effect of diet on the risk of BC, numerous studies are conducted measuring the association between single food items and risk of BC in general^14–17^. Knowing that individuals do not consume nutrients separately, it is important to investigate the effect of our diet on health with a holistic dietary approach rather than individual nutrients especially when evaluating the association of diet and BC risk^18^. As a consequence of the Neolithic and industrial revolutions, the staple foods of the Western diet (WD), such as processed meats, sugar, alcohol, and refined grains, became the main component of the diet of a people^19^. We know for years that the WD is potentially detrimental to our health. For example, the advent of the WD has been associated to an increase in the occurrence of obesity, mortality from heart diseases, type 2 diabetes, hypertension, cancer and other diet-related diseases^20^. It has been suggested that the Iranian population is different from those in the developed world with respect to genetics, lifestyle, diet, and environment^21,22^. However, the rapid demographic change, urbanization, and social development are causing several important health-related changes among the Iranian population, including nutritional transition to a westernised diet and sedentary lifestyle^17^.

The importance of dietary pattern in our health has only recently received attention and evidence on the association of dietary patterns and risk of IDC and ILC is still scarce. Accordingly, a meta-analysis of six studies on the association between a WD and the risk of IDC reported a significant and positive association between WD and risk of IDC^13^. Moreover, combined the results of studies showed a positive association between higher adherence to a WD and risk of ILC^13^. This meta-analysis also highlighted the lack of well-designed large epidemiological studies on this topic and suggested that well-designed studies are required to approve the association of WD and histopathological subtypes of BC.

Given the importance of WD in the aetiology of majority of diseases including cancer, the main body of evidence on diet and BC comes from epidemiologic studies conducted in developed countries. The studies also have mainly focused on single food items approach with less attention to BC subtypes. In the current study, our aim was to assess the potential associations between a WD and the risk of developing IDC and ILC. We further examined if the pattern of association differs by subtype with regard to menopausal status. The observed difference in the impact of WD on BC subtypes might provide new insight in the aetiology of BC.

## Results

### General characteristics

General characteristics, dietary information, and adherence to the WD between controls and IDC cases, and between controls and ILC cases are reported in Table 1. The mean (± standard deviation [SD]) age of controls was 48.74 (± 10.48) and for IDC cases, and ILC patients it was 47.2 ± 9.4 and 50.5 ± 10.3 respectively (p < 0.001). Family history of BC was reported among 14% of control participants, whereas 27% of IDC and 16% of ILC cases reported BC among their family members (p = 0.001). Overall, 36% of control participants, 39% of IDC cases and 41% of ILC cases were post-menopausal (p > 0.05). Also, compared to the control participants (7%), IDC and ILC cases were more likely to be smoker (14% and 15%, respectively) (p < 0.05). The mean (± SD) of the WDS was 24.3 (± 2.5), 23.1 (± 4.1) and 22.4 (± 4.4) for IDC, ILC cases and controls, respectively (p = 0.01) (Table 1). Compared to the control group, the average of consumption of all the WD components was higher among IDC and ILC cases. However, there is no difference in fruit consumption between cases and control group.

**Table 1:** General characteristics, dietary variables, and WD score between controls and IDC cases, and between controls and ILC cases.

	Control	IDC	p-value^1^ IDC vs. control	ILC	p-value^1^ ILC vs. control
**General characteristics**					
Age (mean ± SD)	48.74 ± 10.48	47.2 ± 9.45	0.05	50.5 ± 10.32	0.01
Family history (n%)					
No	867 (85.9)	619 (72.9)	0.001	134 (83.8)	0.02
Second relative	54 (5.8)	70 (8.3)		14 (8.7)	
First relative	88 (8.7)	160 (18.8)		12 (7.5)	
Smoking (n%)					
No	937 (92.9)	724 (85.3)	< 0.001	136 (85.0)	< 0.001
Yes	72 (7.1)	125 (14.7)		24 (15.0)	
OCP use (n%)					
Never	601 (59.6)	455 (53.6)	0.01	82 (51.2)	0.04
Ever	408 (40.4)	394 (46.4)		78 (48.8)	
Chest X-ray history (n%)					
No	317 (31.4)	242 (28.5)	0.17	114 (71.3)	0.001
Yes	692 (68.6)	607 (71.5)		46 (28.7)	
History of benign breast disease (n%)					
No	943 (93.5)	731 (86.1)	0.001	138 (86.3)	0.001
Yes	66 (6.5)	118 (13.9)		22 (13.7)	
Physical activity (n%)					
No	799 (79.2)	683 (80.4)	0.50	132 (82.5)	0.33
Yes	210 (20.8)	166 (19.6)		28 (17.5)	
BMI (n%)					
Normal (18.50–24.99)^	359 (33.2)	270 (29.8)	0.001	359 (33.2)	0.56
overweight (25.00 to 29.99)	489 (48.5)	373 (43.9)		489 (48.5)	
Obese (≥ 30.00)	161 (16.0)	206 (24.3)		161 (16.0)	
Age at first delivery (year) (n%)					
< 18	355 (35.2)	200 (23.6)	0.001	46 (28.8)	0.50
18–23	284 (28.1)	254 (29.9)		52 (32.5)	
24–30	158 (15.7)	142 (16.7)		28 (17.4)	
≥ 31	131 (13.0)	181 (21.3)		22 (13.8)	
Nulliparous	81 (8.0)	72 (8.5)		12 (7.5)	
Breastfeeding (month) (n%)					
0–5	184 (18.2)	204 (24.0)	0.001	30 (18.7)	0.01
6–17	53 (5.3)	72 (8.5)		14 (8.8)	
18–29	128 (12.7)	114 (13.4)		20 (12.5)	
30–41	116 (11.5)	90 (10.6)		18 (11.2)	
≥ 42	528 (52.3)	369 (43.5)		78 (48.8)	
History of miscarriage (n%)					
No	694 (68.8)	567 (66.8)	0.35	94 (58.8)	0.04
Yes	315 (31.2)	282 (33.2)		66 (41.2)	
Menarche age (year) (n%)					
< 12	138 (13.7)	131 (15.4)	0.44	38 (23.7)	
12–13	431 (42.7)	343 (40.4)		64 (40.0)	
≥ 14	440 (43.6)	375 (44.2)		58 (36.3)	
Menopausal status (n%)					
Pre-menopausal	647 (64.1)	518 (61.0)	0.16	94 (58.8)	0.19
Post-menopausal	362 (35.9)	331 (39.0)		66 (41.2)	
**Dietary variables (mean ± SD)**					
WDS	22.42 (4.43)	24.31 (2.47)	0.001	23.12 (4.13)	0.06
Energy intake (kcal per day)	2418.34 ± 602.21	2601.36 ± 608.32	< 0.001	2721.14 ± 732.78	< 0.001
Cream (gram per day)	2.45 (47.78)	3.36 (16.07)	0.001	3.58 (5.59)	< 0.001
Egg (gram per day)	18.05 (15.70)	19.15 (14.98)	0.02	18.24 (15.03)	0.04
Red and processed meet (gram per day)	79.10 (54.07)	83.52 (51.26)	0.001	83.34 (58.24)	0.001
Butter (gram per day)	4.92 (8.62)	12.98 (8.79)	< 0.001	12.78 (7.22)	< 0.001
Margarine (gram per day)	10.88 (15.86)	12.03 (14.27)	0.001	11.56 (14.71)	0.06
Animal fat (gram per day)	0.21 (1.21)	0.27 (0.81)	0.001	0.26 (1.31)	0.001
Pasta (gram per day)	34.75 (51.25)	35.08 (51.23)	0.02	34.94 (47.06)	0.07
Sugar (gram per day)	18.56 (39.56)	18.18 (43.65)	0.01	18.62 (47.08)	0.09
Dressing (gram per day)	6.37 (8.43)	6.51 (9.11)	0.01	6.46 (9.18)	0.01
Dips (gram per day)	5.62 (9.20)	5.69 (8.33)	0.03	5.58 (8.76)	0.01
Vegetables (gram per day)	198.98 (107.23)	203.06 (134.43)	0.001	199.15 (143.60)	0.08
Fruit (gram per day)	133.89 (108.04)	133.61 (109.23)	0.23	133.63	0.34

*IDC* invasive ductal carcinoma, *ILC* invasive lobular carcinoma, *SD* standard deviation, *WDS* Western diet score.

^1^p-value are based on t-test (continuous variables) or X^2^ (categorical variables).

^^^Underweight and normal BMI were merged in one group (due to the small number of underweights).

General characteristics and dietary information based on tertiles of adherence to the Western dietary pattern are presented in Table 2. As shown in Table 2, those in the highest vs. lowest tertile of adherence to WD were more likely to have IDC (52%) and less likely to be control (38%) and ILC (9%) (p < 0.001). Dietary information based on tertiles of adherence to the components of a WD pattern among the control participants and IDC and ILC of the breast are presented in Supplementary Table 1.

**Table 2:** General characteristics, and dietary information based on tertiles of adherence to the Western dietary pattern.

Factors	Tertile of Western diet score			p-value
	Tertile 1	Tertile 2	Tertile 3	
	n (%)	n (%)	n (%)	
**Participants**				
Controls	409 (56.80)	390 (52.14)	210 (38.18)	0.001*
Ductal (IDC)	253 (35.14)	307 (41.04)	289 (52.55)	
Lobular (ILC)	58 (8.06)	51 (6.82)	51 (9.27)	
Age (year)				0.001*
< 40	481 (70.22)	25 (3.24)	23 (4.11)	
41–50	22 (3.21)	632 (81.76)	14 (2.5)	
51–60	74 (10.80)	13 (1.68)	435 (77.68)	
> 60	108 (15.77)	103 (13.32)	88 (15.71)	
Family history of BC				0.001*
No	599 (83.20)	621 (83.02)	400 (72.73)	
Second relative	42 (5.83)	46 (6.15)	50 (9.09)	
First relative^1^	79 (10.97)	81 (10.83)	100 (18.18)	
Smoking				< 0.001*
No	669 (92.92)	646 (86.36)	482 (87.64)	
Yes	51 (7.08)	102 (13.64)	68 (12.36)	
OCP use				0.06*
Never	429 (59.58)	401 (53.61)	308 (56)	
Ever	291 (40.42)	347 (46.39)	242 (44)	
Chest X-ray history				< 0.001*
No	209 (29.03)	232 (31.02)	232 (42.18)	
Yes	511 (70.97)	516 (68.98)	318 (57.82)	
History of benign breast disease				0.61*
No	653 (90.69)	667 (89.17)	492 (89.45)	
Yes	67 (9.31)	81 (10.83)	58 (10.55)	
Physical activity^2^				0.07*
No	593 (82.36)	580 (77.54)	441 (80.18)	
Yes	127 (17.64)	168 (22.46)	109 (19.82)	
BMI				0.08*
Normal (18.50–24.99)	248 (34.44)	224 (29.95)	153 (27.82)	
Underweight (< 18.5)	20 (2.78)	12 (1.60)	12 (2.18)	
Overweight (25.00 to 29.99)	325 (45.14)	355 (47.46)	264 (48)	
Obese (≥ 30.00)	127 (17.64)	157 (20.99)	121 (22)	
Age at first delivery (year)				0.001*
< 18	185 (25.70)	238 (31.82)	178 (32.36)	
18–23	177 (24.58)	233 (31.15)	180 (32.73)	
24–30	136 (18.89)	103 (13.77)	89 (16.18)	
≥ 31	141 (19.58)	123 (16.44)	70 (12.73)	
Nulliparous	81 (11.25)	51 (6.82)	33 (6.0)	
Breastfeeding (month)				0.001*
0–5	198 (27.50)	128 (17.11)	92 (16.73)	
6–17	52 (7.22)	52 (6.95)	35 (6.36)	
18–29	117 (16.25)	89 (11.90)	56 (10.18)	
30–41	81 (11.25)	74 (9.89)	69 (12.55)	
≥ 42	272 (37.78)	405 (54.15)	298 (54.18)	
History of miscarriage				0.003*
No	511 (70.97)	504 (67.38)	340 (61.82)	
Yes	209 (29.03)	244 (32.62)	210 (38.18)	
Menarche age (year)				0.008*
< 12	125 (17.36)	88 (11.76)	94 (17.09)	
12–13	284 (39.44)	340 (45.46)	214 (38.91)	
≥ 14	311 (43.20)	320 (42.78)	242 (44)	
Menopausal status^3^				0.001*
Pre-menopausal	548 (76.11)	555 (74.20)	156 (28.36)	
Post-menopausal	172 (23.89)	193 (25.80)	394 (71.64)	
**Dietary variables**				
Energy intake kcal per day (mean (SD)	2354.41 ± 502.34	2684.23 ± 629.54	2784.03 ± 607.43	< 0.0001**
Cream gram per day (mean ± SD)	1.55 (3.87)	2.36 (4.69)	3.14 (5.47)	< 0.0001**
Egg gram per day (mean ± SD)	10.25 (11.21)	16.29 (14.58)	24.90 (18.40)	< 0.0001**
Red and processed meat gram per day (mean ± SD)	48.21 (42.30)	73.05 (54.61)	118.11 (62.51)	< 0.0001**
Butter gram per day (mean ± SD)	1.80 (5.51)	3.74 (8.01)	6.18 (9.99)	< 0.0001**
Margarine gram per day (mean ± SD)	7.84 (12.93)	11.51 (15.41)	14.85 (17.20)	0.001**
Animal fat gram per day (mean ± SD)	0.02 (0.29)	0.11 (0.91)	0.51 (1.78)	< 0.0001**
Pasta gram per day (mean ± SD)	32.43 (39.75)	32.22 (42.10)	41.62 (65.94)	< 0.0001**
Sugar gram per day (mean ± SD)	10.94 (26.97)	15.81 (43.24)	27.92 (64.32)	< 0.0001**
Dressing gram per day (mean ± SD)	2.80 (6.61)	6.24 (9.66)	10.08 (11.36)	< 0.0001**
Dips gram per day (mean ± SD)	2.99 (6.26)	5.85 (9.06)	8.01 (12.03)	< 0.0001**
Vegetable gram per day (mean ± SD)	184.04 (150.51)	204.76 (141.26)	208.91 (131.48)	< 0.0001**
Fruits gram per day (mean ± SD)	119.94 (111.78)	132.03 (106.63)	149.71 (110.95)	< 0.0001**

*IDC* invasive ductal carcinoma, *ILC* invasive lobular carcinoma, *BC* breast cancer; *SD* standard deviation, *OCP* oral contraceptive pills, *BMI* body mass index.

p values are based on *the Chi-squared test or **one-way ANOVA test.

^1^First or both first and second relatives.

^2^Based on WHO definition: 30 min or more of moderate aerobic activity at least 3 or more times/week on a regular basis.

^3^Only natural menopause.

### Associations between the WD pattern and risk of IDC

The estimated ORs for adherence to the WD pattern in IDC patients are presented in Table 3. Since there were no significant differences between the two models, (model 1: not adjusted for fruit and vegetable and model 2: adjusted for fruit and vegetable), we reported the results of the fully adjusted model 2. Overall, more adherence to the WD pattern was associated with an increased risk of IDC in both model 1 and model 2 (Model 2: OR highest vs. lowest tertile: 2.45, 95% CI 1.88, 3.17). Also, test for a linear trend across the tertiles of WD adherence was significant (p‐trend < 0.001).

**Table 3:** Odds ratios (OR) and 95% confidence intervals (CIs) of breast cancer histological subtypes by adherence to the Western diet score (tertiles).

	Tumour subtype								*p* for heterogeneity^2^ lobular vs. ductal
	IDC				ILC				
	Tertile 1 OR (95% CI)	Tertile 2 OR (95% CI)	Tertile 3 OR (95% CI)	*p* trend	Tertile 1 OR (95% CI)	Tertile 2 OR (95% CI)	Tertile 3 OR (95% CI)	*p* trend	
**All participants**				–					
Crude^1^	1 (reference)	0.24 (0.02, 0.45)	0.79 (0.56, 1.03)	**0.01**	1 (reference)	0.65 (0.21, 1.08)	1.22 (0.77, 1.66)	0.001	0.07
Model 1^a^	1 (reference)	1.34 (1.06, 1.70)	2.44 (1.88, 3.16)	** < 0.001**	1 (reference)	0.84 (0.16, 2.93)	1.70 (0.67, 3.35)	0. 01	0.04
Model 2^b^	1 (reference)	1.33 (1.05, 1.69)	2.45 (1.88, 3.17)	** < 0.001**	1 (reference)	1.06 (0.23, 3.13)	1.63 (0.63, 3.25)	0.06	**0.03**
**Pre-menopause**				–					
Crude	1 (reference)	0.18 (0.06, 0.43)	0.91 (0.52, 1.30)	0.06	1 (reference)	0.88 (0.34, 1.42)	2.03 (1.39, 2.68)	**0.001**	0.12
Model 1^a^	1 (reference)	1.26 (0.96, 1.66)	2.95 (1.91, 4.55)	**0.001**	1 (reference)	2.01 (1.10, 3.62)	5.38 (2.62, 11.03)	** < 0.001**	0.65
Model 2^b^	1 (reference)	1.25 (0.94, 1.64)	2.95 (1.91, 4.56)	** < 0.001**	1 (reference)	2.36 (1.28, 4.33)	5.25 (0.54, 10.82)	**0.03**	**0.02**
**Post-menopause**									
Crude	1 (reference)	0.46 (0.01, 0.92)	0.99 (0.59, 1.40)	0.17	1 (reference)	0.15 (0.01, 0.91)	0.43 (0.02, 1.10)	0.05	0.44
Model 1^a^	1 (reference)	1.20 (0.72, 1.99)	2.14 (1.38, 3.34)	**0.001**	1 (reference)	1.00 (0.43, 2.32)	1.37 (0.65, 2.86)	0.35	**0.02**
Model 2^b^	1 (reference)	1.20 (0.79, 1.99)	2.16 (1.39, 3.37)	** < 0.001**	1 (reference)	1.05 (0.45, 2.45)	1.35 (0.64, 2.85)	0.38	**0.04**

*IDC* invasive ductal carcinoma, *ILC* invasive lobular carcinoma, *OR* odds ratio, *CI* confidence interval.

Bolded p-values represent statistically significant associations (Pp < 0.05).

^1^Multinomial logistic regression.

^2^Using Wald-test of the hypothesis that both subtypes of breast cancer share the same odds ratio for each exposure under study.

^a^Adjusted for energy intake, family history of BC, smoking status, OCP, chest X-ray, history of benign breast disease, BMI, physical activity, age at first delivery (year), breastfeeding (month), history of miscarriage, menarche age (year) and menopausal status.

^b^Adjusted for model 1+ fruit and vegetable intakes.

### Associations between the WD pattern and ILC

As Table 3 shows the estimated ORs for adherence to the WD pattern and ILC breast cancer risk, we found that higher adherence to the WD pattern was not associated with the risk of ILC in both model 1 and model 2 (Model 2: OR highest vs. lowest tertile: 1.63, 95% CI 0.63, 3.25, p-trend = 0.06). Test for heterogeneity comparing the association of adherence to WD and risk of IDC and ILC was also significant (p-heterogeneity = 0.03).

### Stratification analysis

Stratification results based on menopausal status are shown in Table 3. As such, among pre-menopause women more adherence to a WD pattern was associated with an increased risk of IDC (Model 2: OR highest vs. lowest tertile: 2.95, 95% CI 1.91, 4.56; p-trend < 0.001). However, results for ILC are similar to ILD but not statistically significant (p-het = 0.02). Among menopausal women, greater adherence to the WD pattern was associated with an increased risk of IDC (Model 2: OR highest vs. lowest tertile: 2.16, 95% CI 1.39, 3.37) but the results were not statistically significant for ILC (Model 2: OR highest vs. lowest tertile: 1.35, 95% CI 0.64, 2.85) (p-heterogeneity = 0.04).

The findings were also in line with the overall results when we stratified the results by smoking status or BMI, suggesting that apart from smoking status or BMI, higher adherence to a WD is a risk factor for IDC, but not for ILC (data are not shown). No significant interaction between the study variables (including smoking status) and menopausal status was found (p for interaction ≥ 0.05 for all). Besides, there was no significant collinearity between the WD score and any other factors.

## Discussion

Results of the present study indicated a significant heterogeneity in the association of adherence to a WD and risk of IDC and ILC as a positive association was observed between WD and risk of IDC. Overall, WD was associated with 2.45 times increase in the risk of IDC. Results among both pre- and post-menopausal women were in line with the overall findings.

In accordance with our findings, in the pooled result of the only available meta-analysis^13^ on IDC and ILC, found a significant and positive association between the WD and the risk of IDC (RR 1.36, 95% CI 1.18, 1.53). Also, in line with our findings, Ronco et al. based on a case control study reported a more than twofold increase in the risk of IDC among women with a higher adherence to the WD, and the association was linear (p-trend = 0.002)^23^. The results of several other previously published case control studies are also in accordance with our findings, suggesting those with a higher adherence to the WD were more prone to IDC^24–27^. However, in contrast with our findings, the results of a cohort study by Cottet et al. found no significant association between higher adherence to a WD and IDC risk (RR 1.17, 95% CI 0.98–1.40)^28^. Nevertheless, the possibility that the positive association observed in case–control studies might be due to recall bias in case–control studies and/or small sample size of the included case control studies, should be taken in to consideration.

Regarding ILC, there are only two published studies (one cohorts and one case–control) that examined the associations between WD and ILC risk and the results were inconsistent as although the case–control study reported a positive association between WD and risk of ILC (OR 1.36)^25^, the cohort study also found a positive association but this did not reach statistical significance (RR = 1.65)^28^. The results of the only available meta-analysis were based on these two above-mentioned studies on dietary patterns and risk of ILC, revealed a marginally positive association between adherence to the WD and risk of ILC (RR 1.45, 95% CI 1.04, 1.86)^13^. To address the inconsistencies between the results of previous studies, the impacts of some important confounders that might affect the findings of the included studies should take in to consideration. As such, although most previous studies adjusted a large number of confounding factors (i.e.; BMI and smoking) which may potentially confound the association between dietary patterns and BC^29^, their results were not fully adjusted for some potentially important confounders, such as physical activity and reproductive factors. In our study, we addressed these issues, as our analysis included reproductive variables, health behaviours, smoking and physical activity. However, another plausible explanation of the non-significant finding in our study is the much lower sample size for ILC (compare with the other two studies) and therefore insufficient power to detect a significant association.

Regarding the subtypes of BC, the MCC-Spanish (multicase-control study on common tumours in Spain) study suggested that higher adherence to the WD seems to increase BC risk in both premenopausal and postmenopausal women with no difference by subtypes^18^. The results of another meta-analysis showed a possible increase in the risk of BC with a higher adherence to WD^29^. The study also reported that among postmenopausal women a significant association is observed between hormone receptor-positive tumours in the subgroup analyses. However, in contract with our study, the authors did not assess the associations of dietary patterns in histological subtypes of BC^29^. Although a previous study claimed that WD is associated with an increased risk of BC among premenopausal women^30^, we found no significant difference in the association of the WD and BC subtypes between pre- and post-menopausal women. However, it should be taken into account that the present study contained limited participants on both menopausal status and breast subtypes, which could have led to a power issue, and the detection of small size effects.

In our study, Western diet, or unhealthy diet, which is notably characterized by higher intakes of red and processed meat, sugar, and sugar-sweetened products, animal fats, and vegetable-processed fats; including margarine, dressings, and dips, was associated with a higher risk of BC. It is suggested that the *N*-nitroso components of meat might increase the risk of breast carcinoma^31,32^. These detrimental components of the WD, might accelerate the initiation, promotion and progression of cancers through several potential biologic mechanisms, including upsurge cellular oxidative stress and potential increase in DNA damage in different tissues of the breast^33^. It has been suggested that adherence to a WD might alter the composition of the gut microbiota, and in turn, the presence and composition of short-chain fatty acids^34^. The short-chain fatty acids generated by gut microbiota seem to have crucial roles in breast cancer occurrence and progression^35,36^.

During the recent years, researchers are becoming more interested in the effect of dietary patterns and risk of BC, as diet can accommodate the complex interplay of nutrients within our food and body. The available evidence, however, on the associations between dietary patterns and BC risk are inconsistent, which might be related to the fact that dietary patterns have distinct impacts on various BC subtypes. Although previous epidemiological investigations on the relationship between BC and food patterns, hormone status, and menopausal state have shown heterogeneous results^28,37^, to date very few studies have examined the association between WD and risk of different subtypes of BC^13,29,38,39^, and the literature on the association of WD and risk of IDC and ILC is still inconclusive. Hence, taking into account the differences in the risk factors, response to treatment and histopathological differences of BC subtypes^6,7^ and also the impacts of components of the WD on BC subtypes, we conclude that, so far, there is still limited robust evidence available on the effect of WD and its components on the risk of IDC and ILC^40^, hence, further research on the specific relation between WD (and its components) and BC subtypes, and the mechanism behind these associations is warranted.

### Strengths and limitations

To the best of our knowledge, this is one of the first case–control studies from a developing country that compares the associations between adherence to a WD and the risk of ILC and IDC in pre- and postmenopausal women. The results are not prone to survival bias because we only considered newly diagnosed cases (incident cases). Anticipating the presence of recall bias, in order to minimize even more the effect of this possible bias, only cases that responded to the questionnaire within the 6 months following the diagnosis were included. Also, factors of interest in this study are considered to be among those that are generally well remembered, regardless of the status of the participants. Likewise, we adjusted the associations for a wide range of established risk factors of BC. Although, the information bias, as a consequence of self-reporting information on food consumption is a common bias in nutritional studies^41^, the strength and direction of this bias should not be significantly different between cases and controls, suggesting that the impact of information bias on our findings might be minimal. The recruitment of women to the control sample only on the basis of an oral declaration of no breast cancer could also be considered as a limitation to this study. As a result, the confirmatory exams or tests might not be necessary^7^. Likewise, another limitation is the relatively small subgroup of ILC. Another limitation of our study was the lack of information on the status of oestrogen (ER) and progesterone (PR) hormone receptor and human epidermal growth factor receptor 2 (HER2) to perform a stratification analysis by hormone receptor status. Thus, a larger study on the heterogeneity of associations of a WD by BC subtypes, hormone receptor status and menopausal status is recommended.

## Conclusions

In conclusion, our findings showed that WD was significantly associated with an increased risk of IDC but this association was not significant but in the same direction for ILC in premenopausal women. In the present study, the results stratified by the menopausal status were in line with the overall findings. Given the significance and strength of the associations found with the WD pattern, in order to determine which dietary habits should be recommended and which should be avoided to reduce BC risk, it is critical to focus not only on the potential protective effects of the healthy dietary patterns, but also on the harmful components of the Western diet.

## Methods

The method section is in accordance with STROBE (The Strengthening the Reporting of Observational Studies in Epidemiology) statement for reporting observational studies (including case–control studies)^42^.

### Ethics statement

The ethical committee of Shiraz University of Medical Sciences (no.13748) approved the study. The study subjects were informed about the study process and confidentiality of data and provided oral informed consent.

### Informed consent

Literate individuals read and signed informed consent forms, and verbal consent was gathered from illiterate participants. Also, written informed consent obtained from a legal guardian of illiterate participants.

### Study population

Details of the study participants and methodology (including case and control selection criteria) of this study have been described elsewhere^7^. In the previous paper, we assessed the associations of some well-recognized risk factors of BC between IDC and ILC^7^. This case–control study included women who were newly diagnosed (within the 6 months following the diagnosis) with invasive BC and were referred to Motahari breast clinic located in Namazi Hospital (affiliated with the Shiraz University of Medical Sciences). This medical centre is referral in Shiraz (the capital of Fars province), and above 80% of newly diagnosed BC women within the Fars province are registered with this centre for BC cares^3^. All women whose diagnosis of primary invasive BC (IDC or ILC) was histologically confirmed during the research period were invited to participate. Those in the control group were considered cancer-free if they verbally verified that they had no current or previous cancer history (no confirmatory exam or test was mandatory).

### Inclusion and exclusion criteria

**Figure 1:**
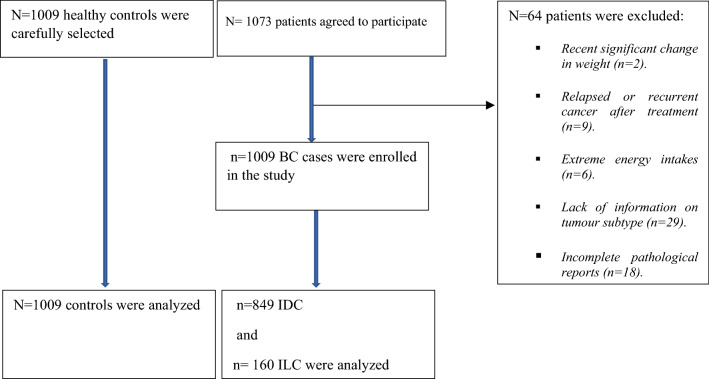
The study follow diagram.

Female patients with newly diagnosed breast cancer, having positive histopathology report on IDC and/or ILC, and also, those who did not followed a prescribed diet regimen by nutritionist were include in the analysis. Patients who experienced a recent significant change in their weight or size from the last 6 months were not included. The latter was done to eliminate the possibility of reverse causation of the association between weight or diet and BC^17^. The case participants were excluded if they had relapsed or recurrent cancer after treatment. Likewise, those with mental disorders or with impaired hearing were excluded^4^. Additionally, participants reporting extreme energy intakes, < 3200 or > 18,000 kJ/day, were excluded in the analysis. A total of 1073 patients (response rate 94%) agreed to participate, of whom, patients were excluded for several reasons including recent significant change in weight (n = 2), relapsed or recurrent cancer after treatment (n = 9), extreme energy intakes (n = 6), lack of information on tumour subtype (n = 29), and incomplete pathological reports (n = 18). After applying the above-mentioned inclusion and exclusion criteria, the study population consisted of 1009 BC cases (849 IDC and 160 ILC) and 1009 healthy controls^7^. The study flow diagram is presented in Fig. 1.

### Data collection

Again, more detail about the method of collecting data on socio-demographic, reproductive and health behaviour of newly diagnosed cases and controls has been reported previously^7^. In addition to the information on dietary consumption, several potential risk factors or confounders including family history of BC, smoking, oral contraceptive pill (OCP), chest X-ray history, history of benign breast disease (BBD), BMI, physical activity, age at first delivery, breastfeeding, history of miscarriage, menarche age, and menopause status were collected^7^.

In this study, premenopausal women were classified as those who had regular menstrual cycles 12 months before to the interview, while postmenopausal women were defined as those who had no menstrual periods in the previous 12 months. Those with no information on menopausal status (nine in the case group and seven in the control group) were classified as premenopausal if they were 47 or under, and postmenopausal if they were 47 or older (the median age of menopause among Iranian women)^43^.

### Dietary intake assessment

Dietary data were collected using a semi-quantitative food frequency questionnaire (FFQ) containing 168 food items with standard serving sizes. The validity and reliability of this questionnaire in an Iranian population has been published previously^44^. Participants were asked to report their consumption frequency of a given serving of each food item daily, weekly, monthly or in a year through an in-person interview with experienced nutritionists. Then, the consumption of frequencies were converted to the daily grams of intake for each food item by using the manual for household measures specialized for Iranians^45^. The nutrient as well as energy intake of participants were then calculated in a daily manner by entering the daily grams of intake of each food item into the Nutritionist-IV software (First Databank; Hearst, San Bruno, CA, USA). This nutrient database is based on the USDA food composition table which is modified for Iranian foods^46^. Then, participants reporting extreme energy intakes, < 3200 or > 18,000 kJ/day, were excluded in the analysis. All food items were categorized into 20 food groups based on the similarity of nutrients or culinary usage of foods^47^.

### Western diet score (WDS)

To test our hypothesis on the association of WD with breast cancer in Iranian population we applied the definition of WD and methods used by the previously published studies on cancer research^47–50^. Briefly, a priori Western dietary pattern was defined based on 8 food groups (i.e., red and processed meats, eggs, animal fat, butter, sugar and sugar products, margarine, dressings, and dips). Based on quintiles of total consumption, a score of 1 to 5 was given to each food item. Those in the lowest quintiles received a score of “1”, while those in the highest quintiles received a score of “5”. The total score for each participant was derived by adding the scores for each dietary item. As a result, the score varied from 8 (the lowest level of adherence) to 40 (the greatest level of adherence) (highest adherence). According to their score, participants were divided into tertiles (low, medium, and high adherence to a Western food pattern). Tertiles were based on distribution in total study population.

### Statistical analysis

The baseline characteristics of the study participants were compared between the WDS tertiles using analysis of variance (ANCOVA) or T-test, for continues variables. Chi-square test was used for categorical variables. Multinomial logistic regression was used to estimate odds ratios (OR) and 95% confidence intervals (CI) of the association between WD and risk of IDC and ILC separately. Based on the multiple logistic models, the p-value for heterogeneity was calculated using Wald test. In the multivariable multinomial logistic regression, models were adjusted for energy intake (kcal per day), fruit and vegetable intake (gram per day), family history of BC, smoking, OCP use, history of chest X-ray, history of BBD, BMI, physical activity, age at first delivery, breastfeeding, history of miscarriage, menarche age, and menopause status. We tested whether associations differed according to subtype by adding an interaction term to the model for WD and subtype. We further performed the stratified analyses based on menopausal status. A post-hoc power analysis suggested that our study had statistical power of 80% to detect associations with OR > 1.65 for ILC and OR > 1.24 for IDC. Based on the adjusted model 2, the p for heterogeneity was calculated using the Wald test. P values for trend were estimated by assigning medians to each category of consumption as a continuous variable. All p-values were two-sided and results were considered to be statistically significant at less than 0.05. All analyses were performed using Stata/SE version 14.2 (Stata Corporation, College Station, TX, USA)^51^.

### Ethics approval and consent to participate

The study was approved by the ethic committee of Shiraz University of Medical Sciences. Informed consent was obtained from all individual participants included in the study. Also, we confirm that all methods were performed in accordance with the declaration of Helsinki.

## Supplementary Information

Supplementary Table 1.

## Data Availability

Datasets that are minimally required to replicate the outcomes of the study will be made available upon reasonable request.

## Abbreviations

DPs: Dietary patterns
WD: Western diet
ORs: Odds ratios
CIs: 95% Confidence intervals
SD: Standard deviation
ANOVA: Analysis of variance
WDS: Western diet score
FFQ: Food frequency questionnaire
IDC: Invasive ductal carcinoma
ILC: Invasive lobular carcinoma
BMI: Body mass index
OCP: Oral contraceptive pills
ER: Oestrogen receptor
PR: Progesterone receptor
HER2: Human epidermal growth factor receptor 2
