# Supplementary material for: Adherence to a western dietary pattern and risk of invasive ductal and lobular breast carcinomas: a case–control study
Source: Sci Rep. 2022 Apr 7;12:5859. doi: 10.1038/s41598-022-09725-5 (PMC8989884; doi:10.1038/s41598-022-09725-5)
Supplement: Supplementary file 1 — Supplementary Table 1. [file 41598_2022_9725_MOESM1_ESM.docx]

**Adherence to a** **Western Dietary Pattern and Risk of Invasive Ductal and Lobular Breast Carcinomas:**

**A Case-Control Study**

**Supplementary Table 1.** Components of the Western dietary pattern, based on tertiles of adherence, among the control participants and invasive ductal carcinoma (IDC) and invasive lobular carcinoma (ILC) of the breast participants.

|  | **Controls** | | | | **IDC** | | | | **ILC** | | | |
| --- | --- | --- | --- | --- | --- | --- | --- | --- | --- | --- | --- | --- |
|  | **Tertile of Western diet score** | | | **P value^1^** | **Tertile of Western diet score** | | | **P value^1^** | **Tertile of Western diet score** | | | **P value^1^** |
|  | **Tertile 1** | **Tertile 2** | **Tertile 3** |  | **Tertile 1** | **Tertile 2** | **Tertile 3** |  | **Tertile 1** | **Tertile 2** | **Tertile 3** |  |
| **Dietary variables (**gram per day (mean ±SD)**)** | | | | |  | | | |  | | | |
| Cream | 1.75 (3.72) | 2.38 (4.45) | 3.18 (5.47) | 0.06 | 2.12 (3.17) | 3.71 (4.72) | 4.24 (5.76) | 0.001 | 1.85 (2.68) | 4.27 (5.34) | 4.65 (5.94) | 0.001 |
| Egg | 11.42 (12.35) | 17.28 (15.24) | 25.44 (19.30) | 0.001 | 12.34 (10.34) | 18.31 (15.03) | 26.82 (19.32) | 0.001 | 11.99 (13.42) | 17.56 (15.06) | 25.16 (18.88) | 0.001 |
| Red and processed meet | 49.08 (43.29) | 70.05 (53.18) | 118.13 (65.98) | 0.001 | 47.25 (32.52) | 83.24 (49.53) | 120.06 (58.61) | 0.001 | 51.57 (48.63) | 79.23 (64.85) | 119.24 (63.48) | 0.001 |
| Butter | 1.98 (6.11) | 4.32 (8.45) | 6.19 (10.01) | 0.001 | 2.12 (4.23) | 3.86 (8.79) | 7.01 (8.28) | 0.001 | 1.92 (5.61) | 4.02 (7.09) | 6.84 (9.34) | 0.001 |
| Margarine | 7.28 (11.85) | 11.51 (15.41) | 13.85 (17.20) | 0.03 | 6.98 (12.85) | 12.08 (14.24) | 15.13 (16.67) | 0.002 | 7.74 (12.35) | 11.93 (15.18) | 15.01 (16.32) | 0.002 |
| Animal fat | 0.02 (0.29) | 0.12 (0.91) | 0.51 (1.78) | 0.01 | 0.03 (0.23) | 0.13 (1.05) | 0.64 (1.86) | 0.001 | 0.02 (0.56) | 0.12 (1.21) | 0.57 (1.89) | 0.001 |
| Pasta | 32.43 (39.75) | 32.22 (42.10) | 39.62 (65.94) | 0.04 | 29.38 (35.45) | 33.28 (43.28) | 42.56 (66.28) | 0.01 | 28.98 (41.58) | 32.90 (43.26) | 42.92 (58.09) | 0.01 |
| Sugar | 11.94 (26.97) | 15.81 (43.24) | 27.92 (64.32) | 0.001 | 9.98 (28.64) | 16.31 (45.21) | 28.27 (65.28) | 0.001 | 10.85 (24.26) | 16.03 (44.52) | 28.99 (65.66) | 0.001 |
| Dressing | 2.80 (6.61) | 6.24 (9.66) | 10.08 (11.36) | 0.001 | 2.20 (6.61) | 7.04 (10.21) | 10.28 (12.68) | 0.001 | 1.99 (7.25) | 6.25 (10.17) | 11.14 (11.51) | 0.001 |
| Dips | 2.99 (6.26) | 5.85 (9.06) | 8.01 (12.03) | 0.001 | 2.90 (6.21) | 6.28 (9.18) | 7.88 (11.78) | 0.001 | 3.05 (7.11) | 5.81 (10.22) | 7.91 (12.54) | 0.001 |
| Vegetables | 184.04 (150.51) | 204.76 (141.26) | 208.91 (131.48) | <0.001 | 192.04 (142.41) | 209.28 (139.89) | 207.88 (130.87) | <0.001 | 183.25 (153.28) | 206.57 (142.32) | 207.62 (139.37) | 0.002 |
| Fruits | 119.94 (111.78) | 132.03 (106.63) | 149.71 (110.95) | <0.001 | 117.58 (110.89) | 133.26 (107.11) | 150.01 (111.54) | <0.001 | 120.03 (112.77) | 133.09 (107.25) | 147.68 (112.66) | <0.001 |

**Abbreviations:** IDC= Invasive ductal carcinoma, ILC= Invasive lobular carcinoma, SD= Standard deviation.

^1^ P value are based on one-way ANOVA test.
